# How commonly do children with cerebral arteriopathy have renovascular disease?

**DOI:** 10.1186/1753-6561-6-S4-O8

**Published:** 2012-07-09

**Authors:** A Willsher, D Roebuck, J Ng, V Ganesan

**Affiliations:** 1 Neurosciences Unit, UCL Institute of Child Health, London, UK; 2 Radiology Department, Great Ormond Street Hospital for Children, London, UK; 3 Neurology Department, Great Ormond Street Hospital for Children, NHS, London, UK

## Introduction

Cerebral arteriopathies, including moyamoya (MM) may be part of a systemic arteriopathy**.** However, patients presenting to neurologists are not routinely evaluated for this. We report the yield of renal angiography in a group of children undergoing catheter cerebral angiography for evaluation of cerebrovascular disease.

## Methods

Children presenting to our neurovascular service and who had had cerebral and renal angiography, other than those with a final diagnosis of vasculitis, were eligible for inclusion. Cerebrovascular disease was categorized MM or other occlusive cerebrovascular disease (OCVD) using standard definitions. Case notes, imaging reports and renal angiograms were retrospectively reviewed. Hypertension (HT) was defined as ≥3 systolic blood pressure (SBP) readings ≥95th percentile.

## Results

Thirty-two children (median age 5.9y, 12 male, 18MM, 14 OCVD) were included. Clinical presentation was arterial ischaemic stroke/transient ischaemic attack in 30, with cerebral infarction in 29. 5 had also clinically presented with hypertension. Renovascular abnormalities were identified in 16 patients - main renal artery (RA) stenosis in 10 (6 OCVD, 4MM), branch RA abnormalities in 5 and peripheral abnormalities in 9. 6 children had multi-site abnormalities. There was no significant difference in the frequency of main RA disease in the MM and OCVD groups (p = 0.21). Of the 27 children with purely neurological presentations; main RA stenosis was seen in 5 and branch/peripheral abnormalities in 10. 5 children had multi-site abnormalities. Based on a median of 5 SBP readings/patient 14 met the definition for HT. Overall BP was poorly recorded and had never been plotted on centile charts in these patients.

## Conclusions

Renovascular abnormalities were common in this group of children with cerebrovascular disease and predominantly neurological presentations. Its clinical correlates were poorly assessed and recorded. Neurologists should be alert to potential systemic involvement and its sequelae in children with cerebral arteriopathy.

